# Bisphenol A Release from Dental Composites and Resin-Modified Glass Ionomers under Two Polymerization Conditions

**DOI:** 10.3390/polym14010046

**Published:** 2021-12-23

**Authors:** Antonin Tichy, Marketa Simkova, Radka Vrbova, Adela Roubickova, Michaela Duskova, Pavel Bradna

**Affiliations:** 1 Institute of Dental Medicine, First Faculty of Medicine, Charles University, General University Hospital, Karlovo Namesti 32, 121 11 Prague, Czech Republic; radka.vrbova@lf1.cuni.cz (R.V.); adela.roubickova@lf1.cuni.cz (A.R.); pavel.bradna@lf1.cuni.cz (P.B.); 2 Institute of Endocrinology, Narodni 8, 116 94 Prague, Czech Republic; mduskova@endo.cz; 3 Department of Natural Compounds, University of Chemistry and Technology, Technicka 6, 166 28 Prague, Czech Republic

**Keywords:** bisphenol A, Bis-GMA, resin composite, glass ionomer cements, light-curing, liquid chromatography, mass spectrometry

## Abstract

Bisphenol A (BPA)-based monomers are commonly contained in dental resin-based materials. As BPA is an endocrine disruptor, its long-term release from restorative composites and resin-modified glass ionomers (RM-GICs) under two polymerization conditions was measured in this study. Specimens of two conventional composites containing BPA-based monomers, two “BPA-free” composites, and two RM-GICs were polymerized from one side for 20 s at 1300 mW/cm^2^ or for 5 s at 3000 mW/cm^2^. The amounts of BPA released in artificial saliva and methanol after 1, 4, 9, 16, 35, 65, 130, and 260 days were measured using liquid chromatography–tandem mass spectrometry. The highest amounts of BPA were released from conventional composites, followed by RM-GICs, while the least was released from “BPA-free” composites. Amounts of released BPA were significantly higher in methanol and decreased gradually after the first day. Fast polymerization (5 s at 3000 mW/cm^2^) resulted in a significantly higher release of BPA after 1 day, but the effect of polymerization conditions was not significant overall. In conclusion, fast polymerization increased the initial release of BPA, but the released amounts were significantly lower than the current tolerable daily intake (4 μg/kg body weight/day) even in methanol, representing the worst-case scenario of BPA release.

## 1. Introduction

Due to its structural similarity with some hormones, bisphenol A (2,2-bis(4-hydroxyphenyl)propane, BPA) is able to bind to various receptors [1,2] and acts as an endocrine disruptor [3]. As a consequence, it has been associated with reproductive, developmental, metabolic, and other disorders [4,5]. However, exposure to BPA is surrounded by controversy. While the U.S. Food and Drug Administration (FDA) and the European Food Safety Authority (EFSA) declared BPA safe at current exposure levels (estimated to be 1.449 μg/kg body weight/day) [6,7], some authors have suggested that adverse effects may occur at doses much below the tolerable daily intake (TDI) [8,9,10,11] of 4 μg/kg b.w. set by the EFSA in 2015 [7].

The use of BPA-based polymers is widespread in food-contact materials and many other products, including dental materials containing BPA-based monomers [12,13], such as bisphenol A glycidyl dimethacrylate (Bis-GMA). As BPA is not intentionally added to dental materials, it is usually present as an impurity from the synthesis of BPA-based monomers or a product of their hydrolytic degradation [14]. Concerns [15] over the release of BPA from dental materials emerged in 1996 after high levels of BPA were detected in the saliva of patients treated with a sealant containing bisphenol A dimethacrylate (Bis-DMA) [16]. These results were disputed [17,18] and numerous following studies reported that the release of BPA from dental materials was significantly lower than TDI [19,20], which led the Scientific Committee on Emerging and Newly Identified Health Risks (SCENIHR) to the conclusion that long-term oral exposure to BPA via dental materials poses only a negligible risk to human health [21]. Nevertheless, due to the apprehension of the low-dose effect [8,9,10,11], BPA-based monomers were excluded from some composites labeled as “BPA-free.”

The release of monomers and other components from resin composites and other resin-based restorative materials, such as resin-modified glass ionomer cements (RM-GICs), is known to be inversely proportional to the degree of conversion [22]. However, the effect of polymerization conditions on the release of BPA has not been sufficiently examined. Manabe et al. reported that the release of BPA from uncured fissure sealants and resin composites in phosphate-buffered saline was significantly higher than if they were polymerized for 60 s [23]. On the other hand, Kwon et al. found that the release of BPA from four resin composites increased with extended irradiation time and reduced distance between the lamp tip and composite surface [24], which was attributed to the photolysis of BPA-based monomers under the high intensity of polymerization light [24]. The effect of irradiation time was also studied by Polydorou et al., but the results were inconclusive as no BPA was detected in most extracts [25]. These ambiguous results suggested that sensitive detection methods are necessary for the trace analysis of released BPA. While the lower limit of quantification (LLOQ) of the liquid chromatography–tandem mass spectrometry (LC–MS/MS) method used by Polydorou et al. was 500 ng/mL [25], the current ultra-high-performance LC–MS/MS (UPLC–MS/MS) methods combined with BPA derivatization reached a LLOQ below 0.1 ng/mL [26,27,28].

The shortening of restorative procedures is one of the targets of contemporary dental material research, and the recent development of high-power polymerization lamps enabled the reduction in irradiation time from 20 s to less than 10 s. Therefore, the objective of this study was to compare the effect of fast polymerization, i.e., irradiation for 5 s at high irradiance, and standard polymerization, i.e., irradiation for 20 s at moderate irradiance, on the long-term release of BPA from various restorative materials measured using an UPLC–MS/MS method with dansyl chloride derivatization [26]. Tested materials included composites containing BPA-based monomers, hereinafter referred to as conventional composites, “BPA-free” composites, and RM-GICs, in which BPA-based monomers were identified in previous studies [29,30]. Artificial saliva, representing the oral environment, and methanol, simulating the worst-case scenario of BPA release, were selected as the extraction media. The null hypotheses tested were (1) that there would be no difference in the release of BPA between fast and standard polymerization, and (2) that there would be no difference in the release of BPA between the tested materials.

## 2. Materials and Methods

Conventional composites Charisma Classic (CC; Kulzer, Hanau, Germany) and Filtek Ultimate Universal Restorative (FU; 3M, St. Paul, MN, USA), “BPA-free” composites Charisma Diamond (CD; Kulzer, Hanau, Germany) and Admira Fusion (AF; Voco, Cuxhaven, Germany), and RM-GICs Photac Fil Quick Aplicap (PF; 3M) and GC Fuji II LC Capsule (F2; GC, Tokyo, Japan) were investigated. According to the material safety data sheets, CC contains Bis-GMA, while FU contains Bis-GMA and ethoxylated Bis-GMA (Bis-EMA). The presence of BPA-based monomers in the examined “BPA-free” composites and RM-GICs was not officially disclosed; however, previous studies identified Bis-EMA in PF [29] and Bis-GMA in F2 [29,30]. The composition of the materials is summarized in Table 1.

### 2.1. Specimen Preparation and Extraction

Polytetrafluoroethylene (PTFE) molds (6 mm diameter, 2 mm thickness) placed on a glass slide were filled with the materials and their surface was flattened using another glass slide. The specimens (surface area 94.2 mm^2^, volume 56.5 mm^3^, mass 0.13–0.16 g) were polymerized from one side using the Valo LED polymerization lamp (Ultradent Products, South Jordan, UT, USA) either for 20 s in “standard” mode (irradiance 1300 mW/cm^2^) or for 5 s in “plasma emulation” mode (3000 mW/cm^2^), which corresponds to radiant exposures of 26 J/cm^2^ and 15 J/cm^2^, respectively. The irradiance in each mode was measured through a glass slide using a USB2000+ spectrometer connected via an optical fiber with a CC-3 cosine corrector. Five measurements per mode were performed after the measuring apparatus had been calibrated with a traceable light source HL-3P-CAL (all Ocean Optics, Dunedin, FL, USA) [31]. The data were processed using SpectraSuite Ocean Optics software.

Thirty minutes after polymerization, specimens were weighed using a digital analytical balance accurate to 0.1 mg and transported to borosilicate glass test tubes with 2 mL of artificial saliva or LC–MS-grade methanol (*n* = 3). The artificial saliva (Hospital laboratory; General University Hospital in Prague, Czech Republic) was prepared by dissolving 0.8 g/L of NaCl, 1.2 g/L of KCl, 0.1 g/L of CaCl_2_.2H_2_O, 0.3 g/L of K_2_HPO_4_.3H_2_O, and 0.1 g/L of MgCl_2_.6H_2_O in distilled water [32] with the pH adjusted to 7.0. The test tubes were closed using screw caps with PTFE-faced septa and incubated at 37 °C in darkness with daily manual agitation. Extracts were collected after 1, 4, 9, 16, 35, 65, 130, and 260 days. When refreshing the extraction media, the test tubes were rinsed 5 times with 0.5 mL of the respective extraction medium, and specimens were carefully removed, weighed using the analytical balance, and replaced in test tubes with 2 mL of fresh extraction medium. To prevent contamination, all instruments, test tubes, and molds had been repeatedly rinsed with methanol.

### 2.2. Uptake of Extraction Media and Mass Loss

After the last extract was collected, the specimens were weighed, dried at room temperature, and repeatedly weighed until a constant mass was obtained. Based on the mass after specimen polymerization (*m*_1_), the highest mass measured during the extraction period (*m*_2_), and the final mass in the dry state (*m*_3_), the uptake of artificial saliva and methanol was calculated as (*m*_2_–*m*_3_)/*m*_1_. Mass loss was calculated using the equation (*m*_1_–*m*_3_)/*m*_1_. The uptake and mass loss were not calculated for RM-GICs, because they inherently contain water, and the results could therefore be misleading.

### 2.3. Chromatographic Analysis

For the LC–MS/MS analysis, BPA and deuterated BPA (d16BPA) standards, dansyl chloride, acetone, sodium bicarbonate, and ammonium formate were purchased from Sigma-Aldrich (St. Louis, MO, USA). LC–MS grade methanol, water for chromatography, and diethylether were obtained from Merck AG (Darmstadt, Germany). Methanol p.a. was purchased from Lach-Ner (Neratovice, Czech Republic). Following the protocol of our previous study [26], a nine-point calibration curve was prepared using 0.032–8.0 ng/mL solutions of BPA in methanol. An amount of 10 µL of the internal standard (d16BPA in methanol) was added to 700 µL of each extract, artificial saliva was extracted using diethylether, and all samples were dried under reduced pressure. The same protocol was used for blanks and control samples with a known addition of BPA. The derivatization of BPA was performed according to [2,33]. Dry residues of the samples were vortexed with 50 µL of dansyl chloride in acetone (1 mg/mL) and 50 µL of a 100 mM sodium bicarbonate buffer, incubated at 50 °C for 15 min and evaporated to dryness. Then, 300 µL of methanol were added and equally diluted with a 10 mM aqueous solution of ammonium formate. An amount of 50 µL of the solution was used for the LC–MS/MS analysis, which was performed using an API 3200 (Sciex, Concord, Canada), a triple-stage quadrupole mass spectrometer with electrospray ionization (ESI) connected to the Eksigent ultra-LC 110 system (Redwood City, CA, USA). Chromatographic separation was performed using a Kinetex C18 1.7 µm (150 × 3.0 mm) column (Phenomenex, Torrance, CA, USA) equipped with a security guard at a flow rate of 0.4 mL/min at 50 °C. A mixture of methanol and water was used as the mobile phase. Further information about LC–MS/MS conditions is available in [2,33]. The LLOQ was 0.042 ng/mL.

### 2.4. Statistical Analysis

The measured amounts of BPA were divided by the specimen mass (*m*_1_) to obtain amounts of BPA released per gram of material (ng/g). To allow for the analysis of the kinetics of BPA release, the average daily release was calculated by dividing the amount of BPA in the extract by the extraction time in days. As eight extracts were prepared from each specimen, the average daily release of BPA from each material in the artificial saliva/methanol was analyzed using two-way repeated measures ANOVA (factors polymerization conditions and extraction time), and pairwise comparisons were performed using Fisher’s LSD test. Cumulative amounts of BPA released from each material over the entire period of 260 days were analyzed using two-way ANOVA (factors extraction medium and polymerization conditions), and materials were compared using t-tests. The uptake of extraction media and mass loss of each tested composite were analyzed analogously to cumulative amounts of released BPA. All analyses were performed at a significance level of 0.05 using Statistica software (version 12.0, TIBCO, Palo Alto, CA, USA).

## 3. Results

### 3.1. Release of BPA

**Figure 1:**
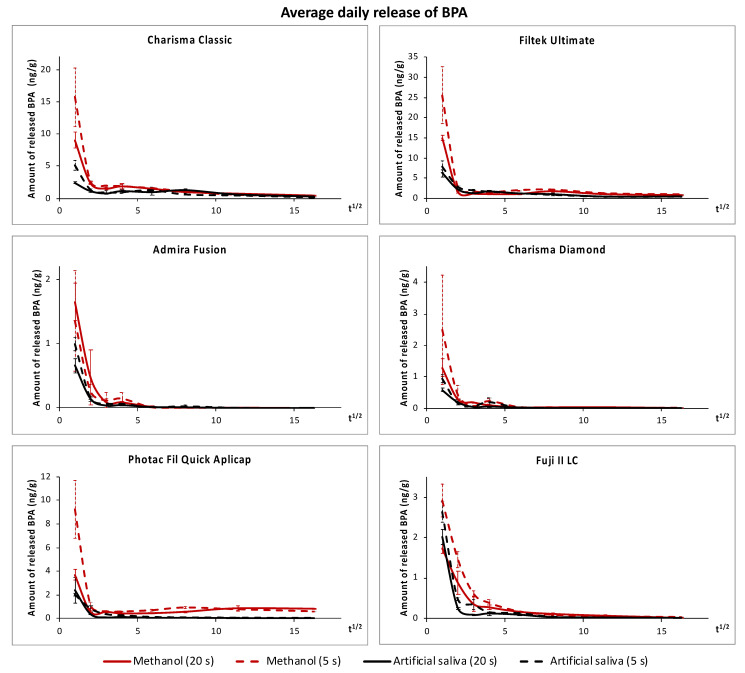
Average daily release of BPA in the artificial saliva (**black**) and methanol (**red**) plotted against the square root of time. The solid lines represent standard polymerization (20 s, 1300 mW/cm^2^), whereas dashed lines represent fast polymerization (5 s, 3000 mW/cm^2^).

The average daily releases of BPA in the artificial saliva and methanol are presented in Table 2 and Table 3, respectively. The kinetics of the release and differences caused by polymerization conditions and extraction media are illustrated in Figure 1. In both extraction media, all materials released the highest amounts of BPA during the first day. The release decreased significantly in the following days (*p* < 0.001) and continued over the entire 260 day period, gradually approaching zero from “BPA-free” composites and the RM-GIC F2. In contrast, the release of BPA from PF in methanol reached its minimum after 35 days, and then it increased slightly.

**Figure 2:**
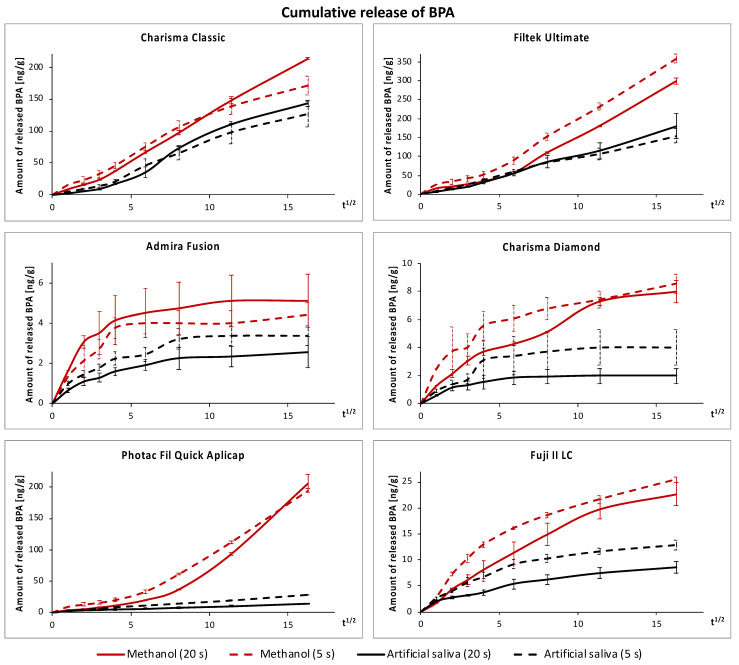
Cumulative amounts of BPA released in the artificial saliva (**black**) and methanol (**red**) plotted against the square root of time. The solid lines represent standard polymerization (20 s, 1300 mW/cm^2^), whereas dashed lines represent fast polymerization (5 s, 3000 mW/cm^2^).

During the first day, significantly more BPA was released from specimens polymerized for 5 s at 3000 mW/cm^2^ compared to those polymerized for 20 s at 1300 mW/cm^2^ (*p* < 0.05), except for PF in the artificial saliva and AF in methanol. However, at longer extraction times, the effect of polymerization conditions on the average daily release of BPA was not significant in most groups (*p* > 0.05). In terms of cumulative release (Table 4, Figure 2), fast polymerization (5 s, 3000 mW/cm^2^) led to a significantly higher release of BPA from RM-GICs in the artificial saliva and FU in methanol than standard polymerization did (20 s, 1300 mW/cm^2^). On the contrary, CC released significantly higher amounts of BPA in methanol if polymerized for 20 s, despite the tendency being initially opposite.

The cumulative data also showed that significantly more BPA was released in methanol (*p* < 0.001). The effect of materials was strongly significant as well (*p* < 0.001). Conventional composites (FU, CC) released substantially more BPA than RM-GICs (PF, F2) (*p* < 0.001), although the cumulative amount of BPA released from PF was similar to CC in methanol (*p* > 0.05). The least BPA was released from the “BPA-free” composites (AF, CD) (*p* < 0.001), regardless of the extraction medium.

### 3.2. Uptake of Extraction Media and Mass Loss

The uptake of artificial saliva was not significantly affected by polymerization conditions (*p* > 0.05), but fast polymerization for 5 s significantly increased the uptake of methanol (*p* < 0.05) in all composites except for CD (Table 5). The mass loss in methanol was significantly higher if composites had been polymerized for 5 s (*p* < 0.05), and a similar tendency was noted in the artificial saliva, but it was not significant (*p* > 0.05) (Table 6).

Both the uptake of extraction media and mass loss were significantly higher in methanol (*p* < 0.001). As for the effect of material, FU exhibited the highest uptake of artificial saliva (*p* < 0.001) and the lowest uptake of methanol (*p* < 0.001), regardless of polymerization conditions. AF had the highest mass loss in the artificial saliva when polymerized for 20 s, but there was no significant difference between the materials if they were polymerized for 5 s. In methanol, the order of materials according to the mass loss was CC > CD > AF > FU (*p* < 0.05).

## 4. Discussion

As silver amalgam is gradually being phased out, restorative dentistry has relied on resin composites and GICs. However, they release unreacted monomers and other substances, which may have adverse effects on human health. Among them, the endocrine-disrupting effect of BPA has drawn particular attention. While the concentrations of BPA in saliva measured by Olea et al. in 1996 [16] were alarming, follow-up studies did not corroborate them [17,18,19]. Moreover, as resin-based materials underwent further development and the sensitivity of analytical methods increased [26,27,28], the reported amounts of released BPA decreased to levels far below the current limits. Nevertheless, as BPA may accumulate in adipose tissue [1,3] and dental materials are listed among the sources of BPA [12], further research on various factors affecting its release is necessary.

The release of various substances from resin composites and RM-GICs is directly affected by the quality of their polymerization [22], which depends on the radiant exitance of the polymerization lamp and irradiation time. As the radiant exitance of modern high-power polymerization lamps may surpass 3000 mW/cm^2^, it allows for the shortening of irradiation time down to 5 s or even less. However, our previous study showed that fast polymerization for 5 s at 3000 mW/cm^2^ was insufficient on the bottom surface of 2 mm thick composite specimens, as opposed to the standard polymerization for 20 s at 1300 mW/cm^2^ [31]. Therefore, the effect of these polymerization conditions on the release of BPA was investigated in this study, as previous reports were inconclusive [23,24,25].

The results of this study revealed that fast polymerization led to a significantly higher release of BPA from most materials during the first day, but polymerization conditions had almost no influence on the cumulative amounts of BPA released over the whole extraction period, so the first null hypothesis was partially rejected. The first-day release of BPA was significantly higher compared to other extraction periods, which agrees with in vivo findings of increased BPA levels in saliva during the first hours after the application of composites or sealants [34,35,36,37,38,39,40]. Initially, BPA was probably extracted from the superficial layers with a lower degree of conversion, mainly from the bottom surface of the specimens, because light energy is significantly attenuated while passing through the materials [31,41]. However, the release of BPA in the oral cavity might be lower, because the deepest layers are often not in contact with saliva. On the other hand, an oxygen-inhibited layer is present on the restoration surface and, if not removed by finishing and polishing, it could increase the release of BPA compared to this in vitro study where oxygen inhibition of polymerization was prevented by polymerizing the specimens through glass slides.

The rate of BPA release decreased significantly after the first day, which agrees with previous studies on the kinetics of BPA release [25,42,43]. This can be attributed to post-irradiation polymerization, which improves the degree of conversion during the first hours/days after irradiation [31,44,45], and the fact that BPA had already been released from the superficial layers. The decrease in BPA release gradually continued toward zero in most materials, and despite using a very sensitive UPLC–MS/MS method, “BPA-free” composites and RM-GICs soon reached levels close to or even below the LLOQ. At such low levels, the reliability of the analysis is decreased, and the experiment was therefore stopped after 260 days even though the release of BPA from CC and FU in both extraction media and PF in methanol was clearly still ongoing even at the end of the 260 day period (Figure 2). In Figure 1, it can be noticed that the gradual decrease in the average daily release was sometimes interrupted by a slight increase. While this could be an irrelevant deviation of the values, a similar phenomenon was observed in a recent study by De Nys et al. who investigated the long-term release of BPA from four resin composites [28]. In aqueous media, the increase could be caused by the hydrolytic degradation of the polymer [28], but further investigation would be necessary to identify the degradation mechanism in methanol.

The effect of polymerization conditions on the release of BPA was most pronounced on the first day. As the radiant exposure, i.e., total light energy delivered to fast-polymerized specimens, was lower, we presume that the lower polymerization degree of the bottom surface [31] contributed to the significantly higher release of BPA from fast-polymerized specimens. In the following periods, there were few significant differences in BPA release between fast and standard polymerization, and as the released amounts were very low, their clinical relevance is disputable. However, the mass loss of all tested composites was higher if they were polymerized for 5 s—significantly in methanol and nonsignificantly in the artificial saliva. This indicates that fast polymerization results in a significantly higher release of various components, presumably due to the lower degree of conversion [22]. In addition, it was revealed that fast polymerization significantly increased the uptake of methanol in all composites except for CD, which also supports this conclusion.

The cumulative data showed that regardless of polymerization conditions, significantly more BPA was released in methanol than in the artificial saliva. Organic alcohols are used to simulate the worst-case scenario of BPA release from resin-based materials, because they are better solvents of methacrylates than water. Consequently, their penetration into hydrophobic composites is faster, and they extract higher amounts of BPA than water or artificial saliva [22]. However, the release of BPA also depends on the monomers used, their purity, and other factors. This was confirmed by the present study, as BPA release was significantly affected by material type, which led to the rejection of the second null hypothesis. Conventional composites released significantly more BPA than other tested materials, because they contain more BPA-based monomers. While Bis-GMA is contained in both tested conventional composites, FU also contains the more hydrophilic Bis-EMA that could contribute to the fact that FU released the highest amounts of BPA and had the highest uptake of artificial saliva. BPA-based monomers were not disclosed in the material safety data sheets of RM-GICs and “BPA-free” composites, but they were identified in extracts of RM-GICs by previous studies [29,30]. The presence of BPA-based monomers and the easier diffusion of extraction media into the structure of RM-GICs presumably caused them to release significantly higher amounts of BPA than “BPA-free” composites. The release of BPA from “BPA-free” composites was slightly surprising, but as only trace amounts were released, it was probably a result of contamination during the manufacturing process.

In comparison with literature data, the amounts of BPA released from conventional composites were similar to the results of other studies using UPLC–MS/MS methods [27,28] but substantially lower than those measured using less sensitive methods [19,20]. To our knowledge, the release of BPA from “BPA-free” composites and RM-GICs was not investigated by other authors. However, regardless of the material type, the amounts of BPA released from the tested materials seem to be a negligible contribution to daily exposure and significantly below the TDI of 4 μg/kg b.w. In the worst-case scenario, i.e., the release from FU polymerized for 5 s in methanol during the first day, the exposure from 1 g of the material would equal 0.009% of the TDI for a 70 kg adult and 0.03% of the TDI for a 20 kg child. It should also be noted that 1 g corresponds to several fillings. Alternatively, when calculated per surface area as advocated by De Nys et al. [28], the exposure from a single crown would equal 0.002–0.004% of the adult’s TDI and 0.008–0.014% of the child’s TDI. In the improbable case of a full-mouth reconstruction with direct composite crowns, the 70 kg adult would be exposed to 0.09% of the TDI, and it must be remembered that the release decreased significantly after the first day.

However, in vitro studies need to be interpreted prudently, because intraoral conditions could affect the release of BPA through factors that are difficult to simulate in vitro, such as the continuous flow of saliva, presence of bacteria and various enzymes, mechanical loading, and changes in temperature and pH. It can also be seen as a limitation that the differences between specimen preparation in vitro and actual filling placement might influence the measured values. Therefore, future studies should investigate the release of BPA in vivo and its implications on human health, as there are many uncertainties especially regarding low-dose adverse effects [8,9,10,11] and the accumulation of BPA [1,3]. The EFSA is currently re-evaluating the evidence of a potential BPA hazard, and the current TDI might change, depending on the updated assessment that is soon to be released. Further research should also be aimed at the development of alternative monomers without a BPA structure.

## 5. Conclusions

Within the limitations of this in vitro study, it can be concluded that fast polymerization significantly increased the initial release of BPA from dental composites and RM-GICs. However, the amounts of BPA released from these materials were substantially lower than the current limits, so they could be considered as a negligible contribution to the daily exposure. On the other hand, no exposure should be dismissed as safe, because the effects of BPA on human health have not been fully clarified to date.

## Figures and Tables

**Table 1:** Composition of materials tested in this study.

Material (Abbreviation)	Manufacturer (Batch Number)	Composition
Charisma Classic A2 (CC)	Kulzer, Hanau, Germany (K010733)	Bis-GMA, TEGDMA, Ba-Al-F glass fillers, pre-polymerized filler, pyrogenic silica, initiator
Filtek Ultimate Universal Restorative A2 Dentin (FU)	3M, St. Paul, MN, USA (N985020)	Bis-GMA, Bis-EMA, UDMA, TEGDMA, PEGDMA, non-agglomerated/non-aggregated silica and zirconia filler, aggregated zirconia/silica cluster filler, initiator
Charisma Diamond A2 (CD)	Kulzer, Hanau, Germany (K010073)	TCD-DI-HEA, UDMA, TEGDMA, Ba-Al-F glass fillers, pyrogenic silica, initiator
Admira Fusion A2 (AF)	Voco, Cuxhaven, Germany (1919447)	no conventional methacrylate monomers, “organically modified ceramics” resin, glass ceramic filler, nano filler, initiator
Photac Fil Quick Aplicap A2 (PF)	3M, St. Paul, MN, USA (4587570)	Na-Ca-Al-La-F silicate glass, HEMA, difunctional monomers, activator (amine), copolymer of acrylic acid and maleic acid, camphorquinone; stabilizers
GC Fuji II LC Capsule A2 (F2)	GC, Tokyo, Japan (190219A)	Al-F silicate glass, polyacrylic acid, HEMA, 2,2,4-trimethyl hexamethylene dicarbonate, TEGDMA

Abbreviations: Bis-GMA—bisphenol A glycidyl dimethacrylate; TEGDMA—triethylene glycol dimethacrylate; Bis-EMA—ethoxylated bisphenol A glycol dimethacrylate; UDMA—urethane dimethacrylate; PEGDM—polyethylene glycol dimethacrylate; TCD-DI-HEA—bis-(acryloyloxymethyl) tricyclodecane; HEMA—2-hydroxyethyl methacrylate.

**Table 2:** Average daily release of BPA in the artificial saliva per gram of material (mean ± SD in ng/g/day).

Material	Polymer Conditions	1 Day (Day 1)	4 Days (Days 2–4)	9 Days (Days 5–9)	16 Days (Days 10–16)	35 Days (Days 17–35)	65 Days (Days 36–65)	130 Days (Days 66–130)	260 Days (Days 130–260)
Charisma Classic	20 s, 1300 mW/cm^2^	2.43 ± 0.19 Aa	1.11 ± 0.12 Abc	0.73 ± 0.02 Acd	1.11 ± 0.20 Abc	0.95 ± 0.38 Abcd	1.27 ± 0.19 Bb	0.57 ± 0.05 Ade	0.25 ± 0.04 Ae
	5 s, 3000 mW/cm^2^	5.09 ± 0.79 Ba	1.36 ± 0.11 Ab	0.97 ± 0.19 Abc	0.99 ± 0.18 Abc	1.31 ± 0.40 Ab	0.64 ± 0.09 Acd	0.50 ± 0.10 Acd	0.21 ± 0.03 Ad
Filtek Ultimate Universal Restorative	20 s, 1300 mW/cm^2^	6.24 ± 0.84 Aa	2.23 ± 0.09 Ab	1.28 ± 0.12 Acd	1.71 ± 0.31 Abc	1.25 ± 0.14 Acd	1.03 ± 0.39 Acd	0.46 ± 0.07 Ad	0.48 ± 0.27 Ad
	5 s, 3000 mW/cm^2^	7.87 ± 1.37 Ba	2.82 ± 0.10 Bb	1.95 ± 0.26 Ac	1.62 ± 0.14 Acd	1.18 ± 0.18 Acd	0.80 ± 0.21 Ade	0.35 ± 0.04 Ae	0.35 ± 0.03 Ae
Charisma Diamond	20 s, 1300 mW/cm^2^	0.58 ± 0.06 Aa	0.19 ± 0.08 Ab	0.03 ± 0.03 Ac	0.05 ± 0.01 Ac	0.02 ± 0.00 Ac	0.00 ± 0.00 Ac	0.00 ± 0.00 Ac	0.00 ± 0.00 Ac
	5 s, 3000 mW/cm^2^	0.93 ± 0.12 Ba	0.15 ± 0.06 Ab	0.07 ± 0.01 Abc	0.10 ± 0.01 Abc	0.01 ± 0.00 Ac	0.01 ± 0.01 Ac	0.00 ± 0.00 Ac	0.00 ± 0.00 Ac
Admira Fusion	20 s, 1300 mW/cm^2^	0.66 ± 0.11 Aa	0.14 ± 0.04 Ab	0.04 ± 0.01 Ac	0.04 ± 0.01 Ac	0.02 ± 0.00 Ac	0.01 ± 0.01 Ac	0.00 ± 0.00 Ac	0.00 ± 0.00 Ac
	5 s, 3000 mW/cm^2^	1.00 ± 0.10 Ba	0.15 ± 0.02 Ab	0.07 ± 0.02 Ac	0.06 ± 0.03 Ac	0.01 ± 0.00 Ac	0.03 ± 0.02 Ac	0.00 ± 0.00 Ac	0.00 ± 0.00 Ac
Photac Fil Quick Aplicap	20 s, 1300 mW/cm^2^	2.40 ± 1.07 Aa	0.37 ± 0.04 Ab	0.09 ± 0.01 Ab	0.13 ± 0.02 Ab	0.06 ± 0.01 Ab	0.06 ± 0.02 Ab	0.03 ± 0.00 Ab	0.03 ± 0.00 Ab
	5 s, 3000 mW/cm^2^	2.02 ± 0.12 Aa	0.84 ± 0.28 Bb	0.37 ± 0.02 Bbc	0.28 ± 0.01 Bbc	0.14 ± 0.01 Bc	0.10 ± 0.01 Ac	0.07 ± 0.01 Bc	0.07 ± 0.01 Bc
GC Fuji II LC Capsule	20 s, 1300 mW/cm^2^	2.01 ± 0.19 Aa	0.25 ± 0.02 Ab	0.09 ± 0.01 Abc	0.12 ± 0.04 Abc	0.09 ± 0.02 Abc	0.03 ± 0.00 Ac	0.02 ± 0.00 Ac	0.01 ± 0.00 Ac
	5 s, 3000 mW/cm^2^	2.62 ± 0.25 Ba	0.46 ± 0.03 Bb	0.34 ± 0.12 Bb	0.15 ± 0.02 Ac	0.13 ± 0.04 Ac	0.04 ± 0.01 Ac	0.02 ± 0.00 Ac	0.01 ± 0.00 Ac

The average daily release was calculated by dividing the amount of BPA in the extract by the extraction time. For example, the extract collected 16 days after specimen preparation contained BPA released between days 10 and 16, so the extraction time was 7 days. Zero values indicate either that the calculated daily release decreased below 0.005 ng/g/day or that it could not be quantified at all, i.e., that the concentration of BPA in the extract was lower than the LLOQ. Significant differences between groups are indicated using letters in the second row of each cell. Different uppercase letters indicate a significant difference between the polymerization conditions; different lowercase letters indicate significant differences between extraction times (in rows).

**Table 3:** Average daily release of BPA in methanol per gram of material (mean ± SD in ng/g/day).

Material	Polymer. Conditions	1 Day (Day 1)	4 Days (Days 2–4)	9 Days (Days 5–9)	16 Days (Days 10–16)	35 Days (Days 17–35)	65 Days (Days 36–65)	130 Days (Days 66–130)	260 Days (Days 130–260)
Charisma Classic	20 s, 1300 mW/cm^2^	9.01 ± 1.23 Aa	2.36 ± 0.19 Ab	1.57 ± 0.17 Ac	1.90 ± 0.44 Abc	1.54 ± 0.07 Acd	1.03 ± 0.07 Ad	0.78 ± 0.06 Be	0.48 ± 0.02 Bf
	5 s, 3000 mW/cm^2^	15.7 ± 4.56 Ba	2.51 ± 0.24 Ab	1.93 ± 0.07 Abc	1.84 ± 0.34 Abc	1.54 ± 0.25 Acd	1.03 ± 0.17 Ad	0.50 ± 0.03 Ae	0.24 ± 0.02 Af
Filtek Ultimate Universal Restorative	20 s, 1300 mW/cm^2^	15.0 ± 0.64 Aa	1.66 ± 0.22 Ab	1.21 ± 0.10 Abc	1.12 ± 0.02 Ac	1.14 ± 0.04 Ac	1.82 ± 0.04 Ab	1.09 ± 0.10 Ac	0.88 ± 0.06 Ac
	5 s, 3000 mW/cm^2^	25.6 ± 7.03 Ba	2.61 ± 0.52 Ab	1.68 ± 0.31 Abc	1.40 ± 0.30 Ac	1.99 ± 0.04 Bb	2.12 ± 0.19 Ab	1.21 ± 0.05 Ac	0.94 ± 0.03 Ad
Charisma Diamond	20 s, 1300 mW/cm^2^	1.28 ± 0.29 Aa	0.28 ± 0.03 Ab	0.18 ± 0.01 Ab	0.09 ± 0.02 Ab	0.06 ± 0.03 Ab	0.03 ± 0.01 Ab	0.03 ± 0.01 Ab	0.01 ± 0.00 Ab
	5 s, 3000 mW/cm^2^	2.50 ± 1.74 Ba	0.41 ± 0.31 Ab	0.06 ± 0.02 Ab	0.22 ± 0.08 Ab	0.03 ± 0.02 Ab	0.02 ± 0.01 Ab	0.01 ± 0.01 Ab	0.01 ± 0.00 Ab
Admira Fusion	20 s, 1300 mW/cm^2^	1.65 ± 0.29 Aa	0.48 ± 0.43 Ab	0.09 ± 0.06 Ab	0.09 ± 0.02 Ab	0.02 ± 0.01 Ab	0.01 ± 0.00 Ab	0.01 ± 0.00 Ab	0.00 ± 0.00 Ab
	5 s, 3000 mW/cm^2^	1.35 ± 0.78 Aa	0.26 ± 0.10 Ab	0.12 ± 0.12 Ab	0.15 ± 0.09 Ab	0.01 ± 0.01 Ab	0.00 ± 0.00 Ab	0.00 ± 0.00 Ab	0.00 ± 0.00 Ab
Photac Fil Quick Aplicap	20 s, 1300 mW/cm^2^	3.71 ± 0.47 Aa	0.56 ± 0.12 Abc	0.57 ± 0.03 Abc	0.43 ± 0.07 Ac	0.45 ± 0.02 Ac	0.57 ± 0.06 Abc	0.87 ± 0.21 Ab	0.83 ± 0.01 Bb
	5 s, 3000 mW/cm^2^	9.27 ± 2.44 Ba	1.11 ± 0.26 Ab	0.62 ± 0.04 Ac	0.60 ± 0.05 Ac	0.70 ± 0.07 Bc	0.94 ± 0.06 Bbc	0.78 ± 0.07 Abc	0.61 ± 0.06 Ac
GC Fuji II LC Capsule	20 s, 1300 mW/cm^2^	1.72 ± 0.11 Aa	0.88 ± 0.29 Ab	0.36 ± 0.18 Ac	0.28 ± 0.06 Acd	0.17 ± 0.01 Acd	0.12 ± 0.01 Acd	0.07 ± 0.01 Acd	0.02 ± 0.00 Ad
	5 s, 3000 mW/cm^2^	2.91 ± 0.41 Ba	1.46 ± 0.19 Bb	0.61 ± 0.09 Ac	0.38 ± 0.08 Acd	0.17 ± 0.02 Ade	0.08 ± 0.01 Ade	0.05 ± 0.01 Ae	0.03 ± 0.01 Ae

The average daily release was calculated by dividing the amount of BPA in the extract by the extraction time. For example, the extract collected 16 days after specimen preparation contained BPA released between days 10 and 16, so the extraction time was 7 days. Zero values indicate either that the calculated daily release decreased below 0.005 ng/g/day or that it could not be quantified at all, i.e., that the concentration of BPA in the extract was lower than the LLOQ. Significant differences between groups are indicated using letters in the second row of each cell. Different uppercase letters indicate a significant difference between the polymerization conditions; different lowercase letters indicate significant differences between extraction times (in rows).

**Table 4:** Cumulative release of BPA per gram of material (mean ± SD in ng/g).

Extraction Medium	Polymerization Conditions	Charisma Classic	Filtek UltimateUniv. Restorative	Charisma Diamond	Admira Fusion	Photac Fil Quick Aplicap	GC Fuji II LC Capsule
Artificial saliva	20 s, 1300 mW/cm^2^	143.7 ± 6.0 Aa	180.4 ± 39.1 Aa	2.09 ± 0.48 Ab	2.54 ± 0.95 Ab	14.1 ± 1.7 Ac	8.84 ± 1.28 Ad
	5 s, 3000 mW/cm^2^	126.4 ± 24.7 Aa	154.1 ± 23.1 Aa	3.34 ± 0.42 Ab	3.39 ± 0.59 ABb	27.6 ± 1.1 Bc	12.9 ± 1.2 Bd
Methanol	20 s, 1300 mW/cm^2^	213.1 ± 4.90 Ba	299.0 ± 18.2 Bb	7.98 ± 0.46 Bc	5.11 ± 1.65 Bd	206.1 ± 17.0 Ca	22.7 ± 3.0 Ce
	5 s, 3000 mW/cm^2^	170.8 ± 19.0 Ca	358.0 ± 16.3 Cb	8.53 ± 0.82 Bc	4.40 ± 1.03 ABd	194.3 ± 7.8 Ca	25.4 ± 2.2 Ce

Cumulative values equal to the total amounts of BPA released from each material over the entire period of 260 days. Significant differences between groups are indicated using letters in the second row of each cell. Different uppercase letters indicate significant differences within each column; different lowercase letters indicate significant differences between the tested materials (in rows).

**Table 5:** The uptake of extraction media by the tested composites (mean ± SD in wt%).

Extraction Medium	Polymerization Conditions	Charisma Classic	Filtek UltimateUniv. Restorative	Charisma Diamond	Admira Fusion
Artificial saliva	20 s, 1300 mW/cm^2^	0.73 ± 0.14 Aa	1.24 ± 0.05 Ab	0.61 ± 0.03 Aa	0.64 ± 0.09 Aa
	5 s, 3000 mW/cm^2^	0.68 ± 0.17 Aa	1.27 ± 0.01 Ab	0.52 ± 0.02 Aa	0.72 ± 0.004 Aa
Methanol	20 s, 1300 mW/cm^2^	3.39 ± 0.15 Ba	1.37 ± 0.05 Bb	3.37 ± 0.11 Ba	2.29 ± 0.11 Bc
	5 s, 3000 mW/cm^2^	3.89 ± 0.17 Ca	1.62 ± 0.17 Cb	3.48 ± 0.01 Bc	2.49 ± 0.07 Cd

Significant differences between groups are indicated using letters in the second row of each cell. Different uppercase letters indicate significant differences within each column; different lowercase letters indicate significant differences between the tested materials (in rows).

**Table 6:** The mass loss of the tested composites (mean ± SD in wt%).

Extraction Medium	Polymerization Conditions	Charisma Classic	Filtek UltimateUniv. Restorative	Charisma Diamond	Admira Fusion
Artificial saliva	20 s, 1300 mW/cm^2^	0.05 ± 0.02 Aa	0.04 ± 0.01 Aa	0.06 ± 0.02 Aa	0.12 ± 0.05 Ab
	5 s, 3000 mW/cm^2^	0.12 ± 0.07 Aa	0.12 ± 0.03 Aa	0.11 ± 0.03 Aa	0.12 ± 0.01 Aa
Methanol	20 s, 1300 mW/cm^2^	2.27 ± 0.10 Ba	0.38 ± 0.06 Bb	2.04 ± 0.08 Bc	1.13 ± 0.08 Bd
	5 s, 3000 mW/cm^2^	3.06 ± 0.19 Ca	0.83 ± 0.13 Cb	2.48 ± 0.05 Cc	1.35 ± 0.08 Cd

Significant differences between groups are indicated using letters in the second row of each cell. Different uppercase letters indicate significant differences within each column; different lowercase letters indicate significant differences between the tested materials (in rows).

## Data Availability

Data available on request from the corresponding author.
